# *BRAF V600E* and *RNF43* Co-mutations Predict Patient Outcomes with Targeted Therapies in Real-World Cases of Colorectal Cancer

**DOI:** 10.1093/oncolo/oyac265

**Published:** 2023-02-13

**Authors:** Julia C F Quintanilha, Ryon P Graf, Geoffrey R Oxnard

**Affiliations:** Clinical Development, Foundation Medicine, Cambridge, MA, USA; Clinical Development, Foundation Medicine, Cambridge, MA, USA; Clinical Development, Foundation Medicine, Cambridge, MA, USA

**Keywords:** BRAF, RNF43, colorectal cancer, predictive biomarker, targeted therapies, real-world data

## Abstract

Anti-BRAF/EGFR therapy is approved for metastatic colorectal cancer (mCRC) with *BRAF*^V600E^ mutations, although not all patients respond. Novel recent findings indicate the potential of *RNF43* mutations to predict outcomes in patients with *BRAF-*mutated microsatellite stable (MSS) mCRC treated with anti-BRAF/EGFR therapy. This study aimed to independently and rapidly validate *BRAF*^V600E^/*RNF43* co-mutations as predictive biomarkers of benefit to anti-EGFR/BRAF therapy. Clinical data were derived from electronic health record data from ~280 US cancer clinics between January 2011 and March 2022 from the Flatiron Health-Foundation Medicine real-world clinico-genomic mCRC database. Real-world cases of *BRAF*^V600E^-mutated mCRC, with patients receiving anti-BRAF/EGFR therapy (*n* = 49), were included. Patients who were MSS, with *RNF43* mutations, had favorable progression-free survival (hazard ratio [HR] 0.29; 95% CI [CI], 0.13-0.65) and overall survival (HR 0.32, 95% CI, 0.12-0.84) compared with wild type. No difference in outcomes was observed between patient groups with *RNF43-*mutant versus wild-type receiving standard-of-care chemotherapy. *BRAF*^V600E^/*RNF43* co*-*mutations predict mCRC anti-BRAF/EGFR outcomes in diverse clinical settings.

## Teaser

This study aimed to validate BRAFV600E/RNF43 co-mutations as predictive biomarkers of benefit to anti-EGFR/BRAF therapy, using clinical data from the Flatiron Health-Foundation Medicine real-world clinico-genomic metastatic colorectal cancer database.

Elez et al^1^ recently showed the novel potential of *RNF43* mutations to predict clinical benefit and favorable outcomes in patients with metastatic microsatellite stable (MSS) colorectal cancer (mCRC) and *BRAF*^V600E^ mutated treated with anti-EGFR/BRAF combinatory regimens. Motivated by the potential of these findings for optimizing the clinical management of mCRC, we sought to leverage the established Flatiron Health-Foundation Medicine (FH-FMI) real-world clinico-genomic database (CGDB) to independently and rapidly validate these findings to help accelerate biomarker development.

As described by Elez et al,^1^*BRAF*^V600E^ mutations are present in approximately 10% of patients with mCRC^2,3^ and are associated with a high rate of mutations in *RNF43*, a tumor suppressor gene involved in the Wnt/β-catenin signaling pathway.^4,5^ Anti-BRAF/EGFR therapy is recommended for patients with mCRC with *BRAF*^V600E^ mutation although not all patients respond. While comprehensive genomic profiling offers an opportunity to study genomic biomarkers of benefit and resistance, adoption in mCRC varies globally.

Elez et al^1^ first studied a discovery cohort consisting of 46 patients treated in a prospective study at a University Hospital in Spain. The authors compared the genomics between responders and non-responders and identified *RNF43* as the top mutated gene in responders. They aimed to validate this finding in an observational cohort of 52 patients from 3 Italian academic centers who received anti-EGFR/BRAF combinatory regimens in second and third lines of therapy. Studying next-generation sequencing results, those with *RNF43* mutation had favorable progression-free survival (PFS) and overall survival (OS) compared to patients with *RNF43* wild type. When grouping by *RNF43* and MSI status, patients with *RNF43* mutations and MSS had more favorable outcomes. We validated the findings of Elez et al^1^ by applying a similar methodology in a real-world cohort of 49 *BRAF*^V600E^ patients with mCRC treated with anti-BRAF/EGFR (encorafenib with cetuximab or panitumumab ± binimetinib) in second or third lines, mostly from non-academic, community practice settings, in the US.

**Figure 1.**
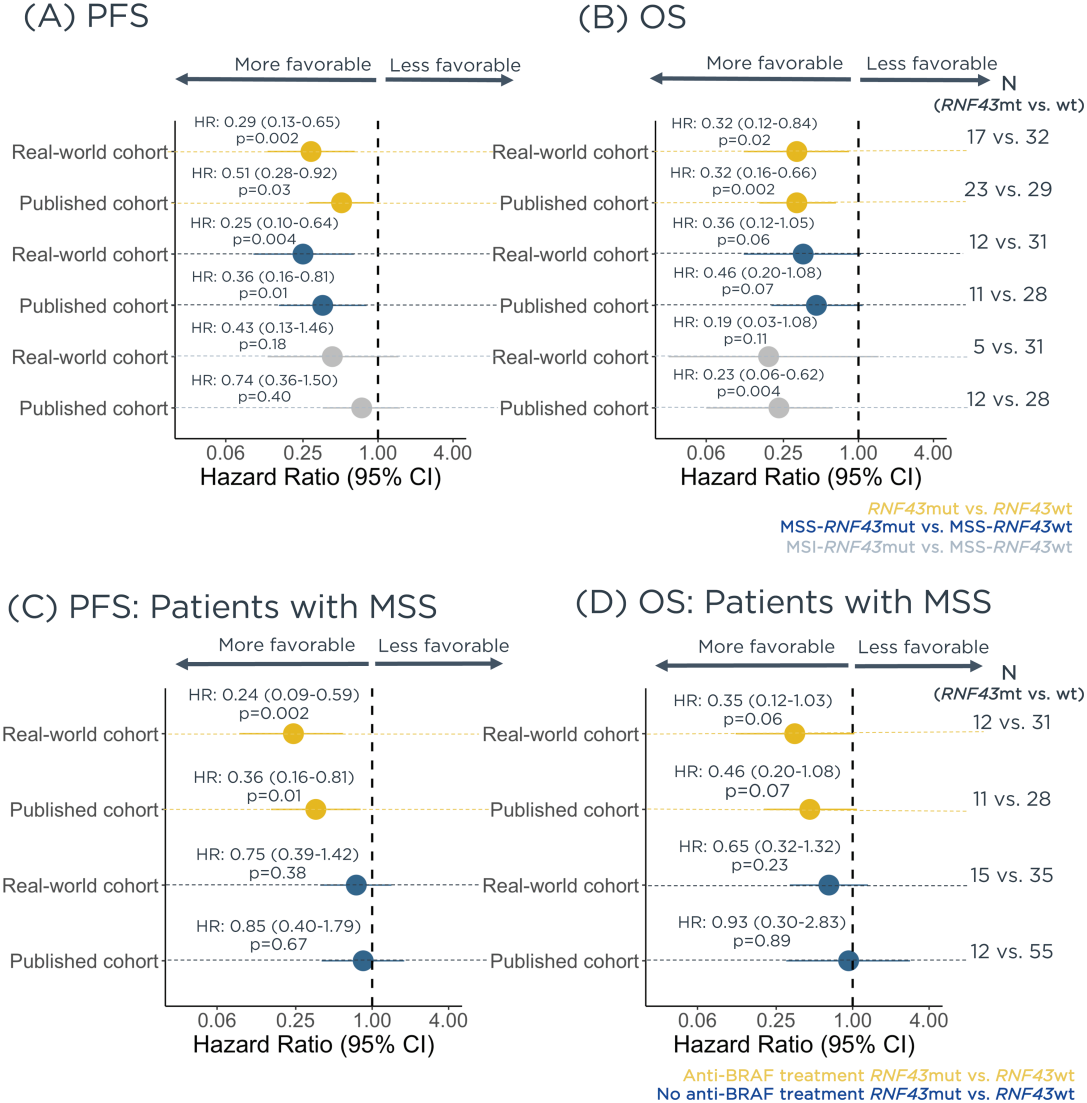
Real-world mCRC patients receiving second and third lines of anti-BRAF/EGFR combinatory regimens have different outcomes according to *RNF43* and MSI status, consistent with Elez et al. (published cohort). Forest plots showing outcomes of all patients treated with anti-BRAF by *RNF43* and MSI status for (**A**) PFS and (**B**) OS. Forest plots showing outcomes of MSS patients treated with anti-BRAF and no anti-BRAF by *RNF43* status for (**C**) PFS and (**D**) OS.

Retrospective longitudinal clinical data were derived from electronic health record data, comprising patient-level structured and unstructured data, curated via technology-enabled abstraction, and were linked to genomic data derived from FMI comprehensive genomic profiling tests in the FH-FMI CGDB by de-identified, deterministic matching, originating from ~280 US cancer clinics between January/2011 and March/2022.^6^ Patients’ baseline characteristics are shown in Table 1, as well as the prevalence of mutations in other genes of the Wnt pathway. Differences in real-world (rw)PFS and real-world (rw)OS were evaluated with the log-rank test and Cox proportional hazard models. To adjust for potential confounders, baseline clinical risk score was estimated from known prognostic features including line of therapy, age at treatment start, gender, race, recurrent disease versus new diagnosis, ECOG status, practice type (academic or community), primary tumor location, albumin, alkaline phosphatase, serum creatinine, hemoglobin, lactate dehydrogenase, neutrophil-to-lymphocyte ratio, platelet, opioid pre-therapy, and steroid pre-therapy. Extended methods can be found in the Supplementary Matierial. Consistent with Elez et al,^1^*BRAF*^V600E^ mCRC patients have favorable rwPFS and rwOS when treated with anti-EGFR/BRAF combinatory regimens. Figure 1A and B shows the comparison between PFS and OS, respectively, obtained from our real-world cohort and the published cohort reported by Elez et al.^1^Supplementary Fig. S1 shows the Kaplan-Meier plots reporting both unadjusted and adjusted results.

**Table 1.** Clinical characteristics of mCRC patients treated with anti-BRAF/EGFR combinatory regimens in 2^nd^ or third lines in CGDB by *RNF43* and MSI status.

	Study population (*n* = 49)	*RNF43* mut, MSS (*n* = 12)	*RNF43* wt, MSS (*n* = 31)	R*NF43* mut, MSI-H (*n* = 5)
Age (years)				
<70	46 (93.9%)	11 (91.7%)	29 (93.5%)	5 (100.0%)
≥70	3 (6.1%)	1 (8.3%)	2 (6.5%)	0
Gender				
Female	27 (55.1%)	8 (66.7%)	13 (41.9%)	5 (100.0%)
Male	22 (44.9%)	4 (33.3%)	18 (58.1%)	0
*ECOG*				
0	20 (42.6%)	6 (50.0%)	13 (44.8%)	1 (20.0%)
1-3	27 (47.4%)	6 (50.0%)	16 (55.2%)	4 (80.0%)
N-miss	2	0	2	0
Primary tumor location				
Left colon/rectum	15 (30.6%)	3 (25.0%)	12 (38.7%)	0
Right colon	22 (44.9%)	7 (58.3%)	12 (38.7%)	2 (40.0%)
NOS	12 (24.5%)	2 (16.7%)	7 (22.6%)	3 (60.0%)
Treatment line				
Second	34 (69.4%)	8 (66.7%)	21 (67.7%)	4 (80.0%)
Third	15 (30.6%)	4 (33.3%)	10 (32.3%)	1 (20.0%)
Combinatory treatment				
Doublet	37 (75.5%)	9 (75.0%)	22 (71.0%)	5 (100.0%)
Triplet	12 (24.5%)	3 (25.0%)	9 (29.0%)	0
Received ICI				
Yes	7 (14.3%)	1 (8.3%)	2 (6.5%)	3 (60.0%)
No	42 (85.7%)	11 (91.7%)	29 (93.5%)	2 (40.0%)
Mutation in other genes of the Wnt pathway				
*AMER1*	3 (6.1%)	0	1 (3.2%)	2 (6.4%)
*APC*	15 (30.6%)	0	15 (48.4%)	0
*AXIN1*	0	0	0	0
*CTNNB1*	0	0	0	0

Abbreviations: CGDB: clinic-genomics database; Doublet: encorafenib with cetuximab or panitumumab; ECOG: Eastern Cooperative Oncology Group; ICI: immune checkpoint inhibitor; mCRC: metastatic colorectal cancer; MSI-H: microsatellite instability high; MSS: microsatellite stable; mut: mutation; NOS: not otherwise specified; Triplet: encorafenib with cetuximab or panitumumab ± binimetinib; wt: wild type.

Elez et al^1^ next confirmed the predictive effect of *RNF43* mutations in patients. They compared outcomes in mCRC MSS patients receiving standard of care chemotherapy ± anti-VEGF and no difference was observed between patients with *RNF43* mutation and wild type. Asking the same question, we compared rwPFS and rwOS of MSS *BRAF*^V600E^ mutated patients receiving chemotherapy (FOLFOX, FOLFIRI, FOLFIXIRI, and CAPEOX) ± anti-VEGF in second and third lines of therapy by *RNF43* status in CGDB, and no difference between those *RNF43* mutated and wild-type was observed. Figure 1C and D shows the comparison between rwPFS and rwOS, respectively, obtained from our real-world cohort and the published cohort reported in Elez et al^1^ for patients treated with anti-BRAF and no anti-BRAF treatment. Supplementary Fig. S2 shows the Kaplan-Meier plots reporting both unadjusted and adjusted results.

This present study demonstrates the potential to use clinico-genomic databases to rapidly validate findings from investigators at academic institutions, contributing to translational efforts in accelerating the use of biomarkers in treatment decisions. The treatment landscape of mCRC has expanded dramatically over the last few years and several treatment options are available for patients with different biomarkers such as MSI, *KRAS*/*BRAF* mutations, and *HER2* amplification. However, even in the subpopulations with such biomarkers, response rates are highly variable with groups of patients lacking benefits, making the treatment course challenging. Studies such as this can help identify which patients are most likely to benefit, especially useful when there are many options. MSS mCRC patients with *BRAF*^V600E^ mutation represent the molecular subgroup with the worst prognosis, characterized by low-immune-reactivity tumors (“immune-cold”), and this novel biomarker can directly improve the management of these patients.^7^

This study provides additional evidence that *BRAF*^V600E^ and *RNF43* co-mutations are biomarkers of anti-BRAF/EGFR effectiveness and reiterates the implications stated by Elez et al^1^ study, in which they suggest the incorporation of *RNF43* as a routine biomarker to inform treatment decisions on the course of mCRC. In addition, Elez et al^1^ highlight the crosstalk of MAPK and RNF43-Wnt pathways during therapy with anti-BRAF/EGFR, indicating future potential therapeutic target approaches.

This study has limitations. There was a relatively small sample size, and although main analyses did not have quantifiable imbalances, and we additionally adjusted for prognostic imbalances, unknown confounders may remain.

In conclusion, we have further provided evidence that *RNF43* mutations might be predictive of anti-BRAF/EGFR treatment outcomes in mCRC in diverse clinical settings, academic and community, from both Europe and the US. Real-world clinico-genomic databases can be a useful tool to rapidly validate novel genomic outcome associations in oncology.

## Supplementary Material

oyac265_suppl_Supplementary_Figure_S1

oyac265_suppl_Supplementary_Figure_S2

oyac265_suppl_Supplementary_Material

oyac265_suppl_Supplementary_Figure_Captions

## Data Availability

The data supporting the findings of this study originated from Flatiron Health, Inc. and Foundation Medicine, Inc. These de-identified data may be made available upon request, and are subject to a license agreement with Flatiron Health and Foundation Medicine; interested researchers should contact cgdb-fmi@flatiron.com and dataaccess@flatiron.com to determine licensing terms. Code is available to bona fide researchers for reasonable request.
