# Supplementary figures and images for: BRAF V600E and RNF43 Co-mutations Predict Patient Outcomes with Targeted Therapies in Real-World Cases of Colorectal Cancer
Source: Oncologist. 2023 Feb 13;28(3):e171–4. doi: 10.1093/oncolo/oyac265 (PMC10020799; doi:10.1093/oncolo/oyac265)

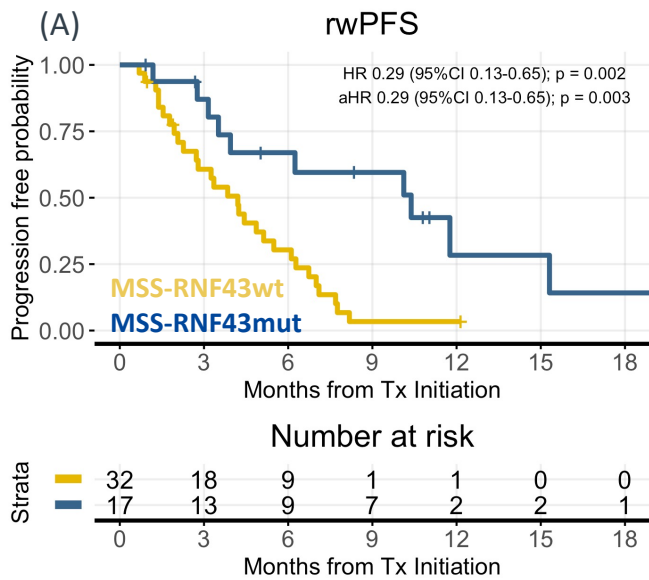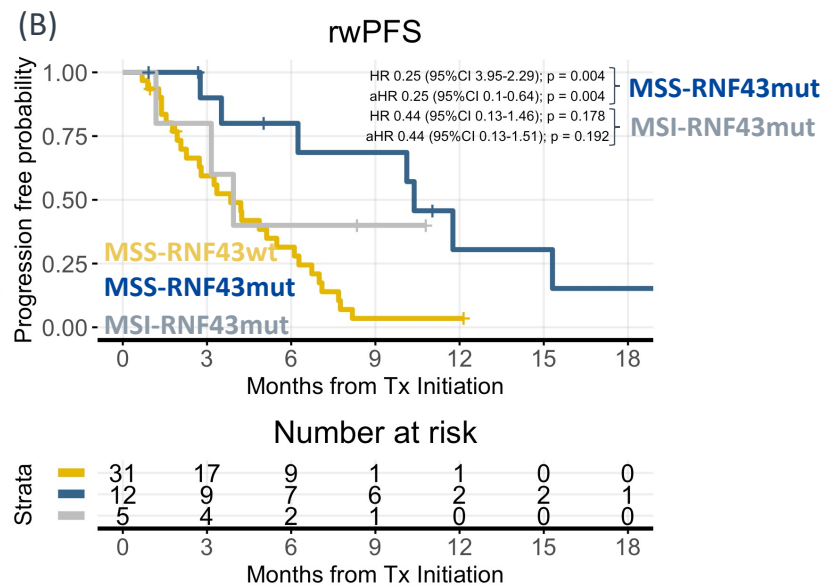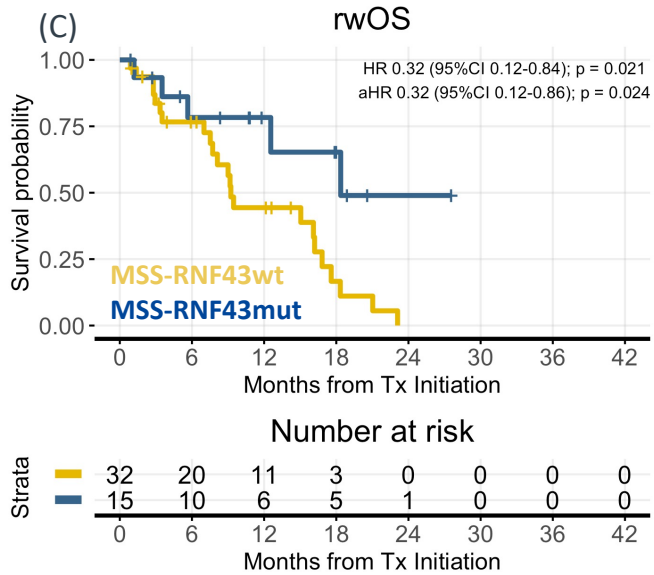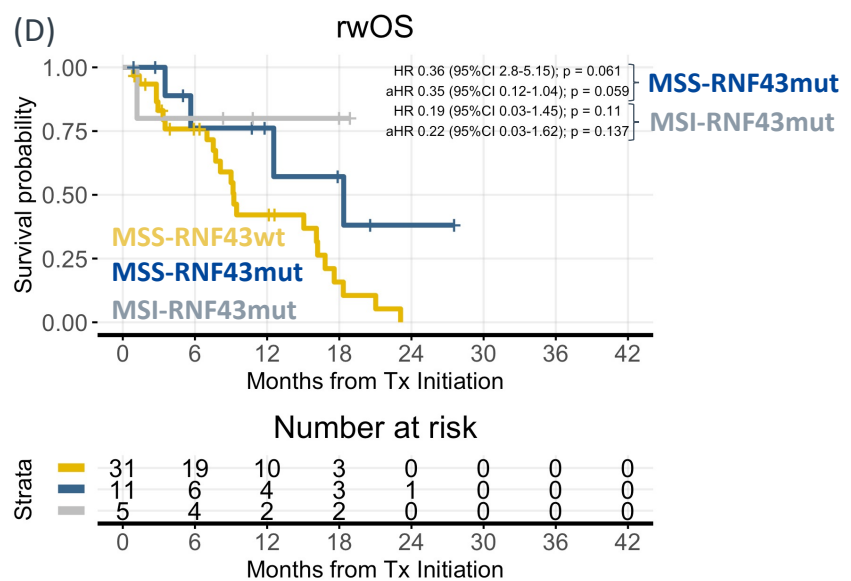

Supplement: oyac265_suppl_Supplementary_Figure_S1 [file oyac265_suppl_supplementary_figure_s1.pdf]

Anti-BRAF treatment

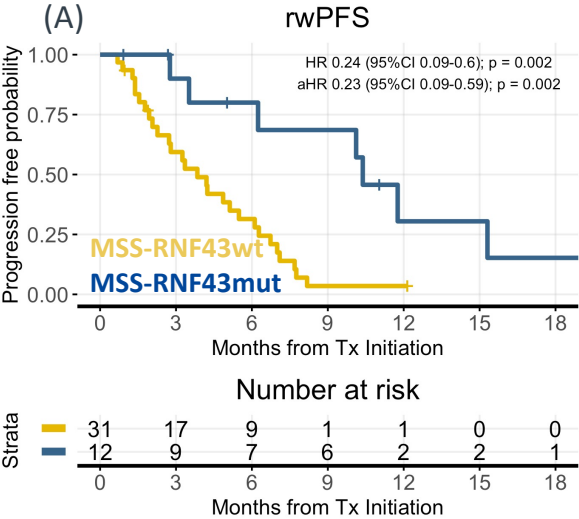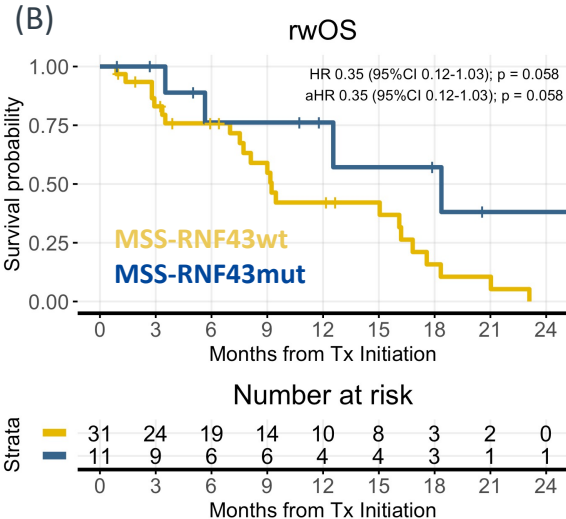

No anti-BRAF treatment

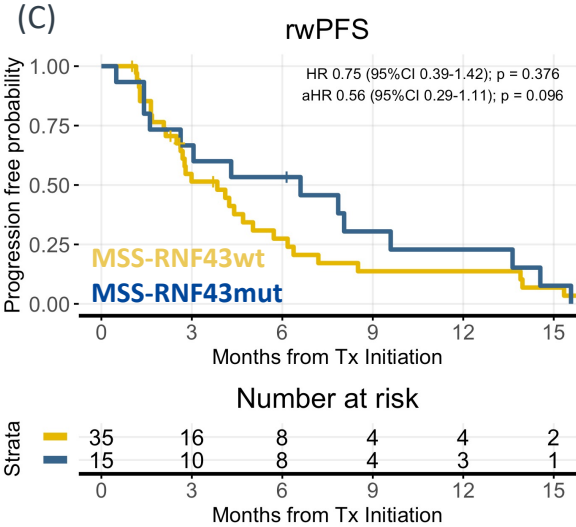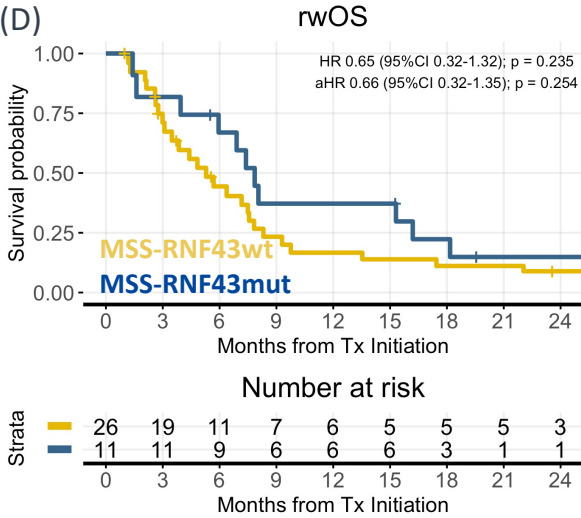

Supplement: oyac265_suppl_Supplementary_Figure_S2 [file oyac265_suppl_supplementary_figure_s2.pdf]
