# Supplementary material for: BRAF V600E and RNF43 Co-mutations Predict Patient Outcomes with Targeted Therapies in Real-World Cases of Colorectal Cancer
Source: Oncologist. 2023 Feb 13;28(3):e171–4. doi: 10.1093/oncolo/oyac265 (PMC10020799; doi:10.1093/oncolo/oyac265)
Supplement: oyac265_suppl_Supplementary_Material [file oyac265_suppl_supplementary_material.docx]

***BRAF*^V600E^ *and RNF43 co-mutations predict outcomes to targeted therapies in real-world colorectal cancer patients***

Julia C. F. Quintanilha^1^, Ryon P. Graf^1^, Geoffrey R. Oxnard^1^

^1^Foundation Medicine, Cambridge, Massachusetts

**Supplementary Methods**

**Outcomes measurement**

Real-world progression-free survival (rwPFS) was calculated from the treatment start date until the time of disease progression or death, and patients not yet reaching progression or death were right-censored at the date of the last clinical visit, laboratory results, or medication order. Real-world overall survival (rwOS) was calculated from the start of treatment to death from any cause, and patients with no record of mortality were right-censored at the date of the last clinic visit or structured activity. rwOS risk intervals were left truncated to the date of the CGP report to account for immortal time, as patients cannot enter the database until a comprehensive genomic profile report is provided^1,2^.

The mortality information in the Flatiron Health database is a composite derived from documents within the EHR, Social Security Death Index, and a commercial death dataset mining data from obituaries and funeral homes. This mortality information has been externally validated in comparison to the National Death Index with > 90% accuracy^3^.

**Risk score generation**

To adjust for potential confounders, baseline clinical risk score was estimated from known prognostic features as the linear predictor from a Cox proportional hazards model for rwOS in patients treated with any eligible line of chemotherapy (FOLFOX, FOLFIRI, FOLFOXIRI/FOLFIRINOX, or CAPOX). The prognostic features included lines of therapy, age at treatment start, gender, race, recurrent disease versus new diagnosis, ECOG status, practice type (academic or community), primary tumor location, albumin, alkaline phosphatase, serum creatinine, hemoglobin, lactate dehydrogenase (LDH), neutrophil-to-lymphocyte ratio (NLR), platelet, opioid pre-therapy, and steroid pre-therapy. The risk scores were estimated as the mean of predicted values from 3 iterations of missing forest imputation. Out-of-sample predictions were obtained by k-fold cross-validation from each imputed data set. The Figure below illustrates the method described.

**
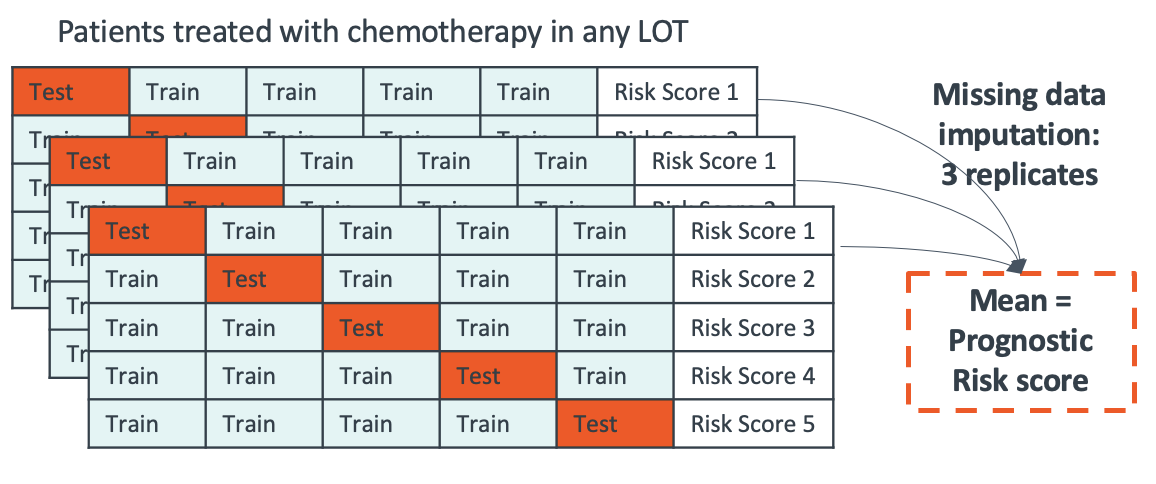
**

**Method for prognostic risk score generation.** Caption: LOT: lines of therapy.

**References**

1. McGough SF, Incerti D, Lyalina S, et al: Penalized regression for left-truncated and right-censored survival data. Stat Med, 2021

2. Brown S, Lavery JA, Shen R, et al: Implications of Selection Bias Due to Delayed Study Entry in Clinical Genomic Studies. JAMA Oncology, 2021

3. Zhang Q, Gossai A, Monroe S, et al: Validation analysis of a composite real-world mortality endpoint for patients with cancer in the United States. Health Services Research n/a.
