# Supplementary material for: BRAF V600E and RNF43 Co-mutations Predict Patient Outcomes with Targeted Therapies in Real-World Cases of Colorectal Cancer
Source: Oncologist. 2023 Feb 13;28(3):e171–4. doi: 10.1093/oncolo/oyac265 (PMC10020799; doi:10.1093/oncolo/oyac265)
Supplement: oyac265_suppl_Supplementary_Figure_Captions [file oyac265_suppl_supplementary_figure_captions.docx]

**Supplementary Figure Captions**

**Supplemental Figure 1.** Real-world mCRC patients receiving 2^nd^ and 3^rd^ lines of BRAF/EGFR inhibitors have different outcomes according to *RNF43* and MSI status, consistent with Elez et al. Kaplan-Meier plots showing outcomes by *RNF43* status for (A) rwPFS and (B) rwOS. Kaplan-Meier plots showing outcomes by MSI and *RNF43* status for (C) rwPFS and (B) rwOS.

**Supplemental Figure 2.** Real-world mCRC MSS patients receiving 2^nd^ and 3^rd^ lines of anti-BRAF/EGFR combinatory regimens have more favorable outcomes when *RNF43* mutant, while no statistical difference in outcomes is observed for patients receiving chemotherapy ± VEGF inhibitors (no anti-BRAF treatment), consistent with Elez et al. Kaplan-Meier plots for patients receiving BRAF/EGFR inhibitors by *RNF43* status for (A) rwPFS and (B) rwOS. Kaplan-Meier plots for patients receiving chemotherapy ± VEGF inhibitors by *RNF43* status for (A) rwPFS and (B) rwOS.
